# *APOE4* impairs autophagy and Aβ clearance by microglial cells

**DOI:** 10.1007/s00011-025-02016-5

**Published:** 2025-04-01

**Authors:** Rawan Bassal, Maria Rivkin-Natan, Alon Rabinovich, Daniel Moris Michaelson, Dan Frenkel, Ronit Pinkas-Kramarski

**Affiliations:** 1 School of Neurobiology, Biochemistry and Biophysics, Tel-Aviv University, Ramat-Aviv, 69978 Israel; 2 Sagol School of Neuroscience, Tel Aviv University, Tel Aviv, Israel

**Keywords:** Alzheimer's disease (AD), Amyloid β, Apolipoprotein E4 (apoE4), Autophagy

## Abstract

Alzheimer’s disease (AD) is a predominant form of dementia in elderly. In sporadic AD and in families with higher risk of AD, correlation with apolipoprotein E4 *(APOE)* allele expression has been found. How *APOE4* induces its pathological effects is still unclear. Several studies indicate that autophagy, a major degradation pathway trough the lysosome, may be compromised in AD. Here we studied, the effects of *APOE* isoforms expression in microglia cells. By using an in-situ model, the clearance of Aβ plaques from brain sections of transgenic 5xFAD mice by the *APOE* expressing microglia was examined. The results show that APOE4 microglia has Impairment In clearance of insoluble Aβ plaques as compared to *APOE3* and *APOE2* microglia. Furthermore, APOE4 affect the uptake of soluble Aβ. We found that microglia expressing *APOE4* exhibit reduced autophagic flux as compared to those expressing *APOE3*. The autophagy inhibitor chloroquine also blocked Aβ plaque uptake in APOE3 expressing cells. Furthermore, we found that *APOE4* expressing microglia have altered mitochondrial dynamics protein expression, mitochondrial morphology and mitochondrial activity compared to those expressing *APOE2*, and *APOE3*. Rapamycin treatment corrected Mitochondrial Membrane Potential in *APOE4-*expressing cells. Taken together, these findings suggest that *APOE4* impairs the activation of autophagy, mitophagy, and Aβ clearance and that autophagy-inducing treatments, such as rapamycin, can enhance autophagy and mitochondrial functions in *APOE4* expressing microglia. Our results reveal a direct link between *APOE4* to autophagy activity in microglia, suggesting that the pathological effects of *APOE4* could be counteracted by pharmacological treatments inducing autophagy, such as rapamycin.

**Supplementary Information:**

The online version contains supplementary material available at 10.1007/s00011-025-02016-5.

## Introduction

Alzheimer’s disease (AD), one of the widespread form of dementia in elderly, is characterized by cognitive decline, presence of senile plaques, neurofibrillary tangles (NFT) and synapse and neuronal loss in the brain [1–3]. Among the risk factors for the disease is the expression of the ε4 allele of apolipoprotein E *(APOE)* in families with late onset and sporadic AD. *APOE4* is strongly associated with increased amyloid-beta (Aβ) deposition and neuronal degeneration [4].

Autophagy is one of the cellular mechanisms which are considered to mediate the clearance of Aβ plaques and NFT [5, 6]. It is an intracellular degradation pathway involved in the turnover of proteins and organelles (normal or dysfunctional) [7]. Results associating *APOE4* to autophagy are limited, however, they might suggest new insights into *APOE4*-mediated pathology. It was shown that AD patients carrying two alleles of *APOE4* express lower mRNA levels of the autophagic and lysosomal proteins (LC3, p62 and LAMP2), compared with *APOE3* patients [8]. Also, it has been shown that autophagy is reduced in *APOE4* murine astrocytes compared to those expressing *AOPE3* [9]. It was also shown that LC3, p62 and LAMP2 mRNA levels were upregulated in *APOE3*-expressing but not *APOE4-*expressing human glioblastoma cells (T98G), following autophagy induction [8]. Additionally, astrocytes expressing *APOE4*, were impaired in Aβ uptake and in clearance of insoluble Aβ plaques [9]. These results suggested a role for autophagy in Aβ removal by astrocytes. It was recently reported that *APOE* plays a major role in microglia maturation and activity [10]. Also, it was shown that autophagy is activated in microglia surrounding amyloid plaques in AD mouse models [11]. Thus, we decided to further investigate the effect of *APOE4* on autophagy in microglia, which may also be involved in Aβ plaques clearance. Indeed, in the present study, we investigated the effects of APOE allele expression on autophagy in microglia under normal and stimulating conditions. The results demonstrated that autophagy is compromised in *APOE4* expressing microglial cells, and that the clearance of Aβ and senile plaques by *APOE4* expressing microglia is impaired. The results also suggest that mitochondrial functions are impaired in *APOE4* microglia and that induction of autophagy/mitophagy by, for example, treatments with mTOR inhibitor (rapamycin) may partially correct the impairment.

## Materials and methods

### Mice

5XFAD mice ( #034848-JAX) were received from Jax. We used brains isolated from 18-month-old male 5XFAD mice (*n* = 2). Brain sections were collected following euthanasia. For euthanasia mice were placed for 5 min in a chamber with CO2 at a rate of change of 50% of the chamber volume per minute. All animal experiments used were in accordance with approval of the Tel Aviv University Animal Care Committee (TAU − 2 - IL − 2111 − 102–3).

### Materials

Antibodies were obtained from the following sources: monoclonal mouse anti-actin (MP Biomedicals, Santa Ana, CA; 691001), polyclonal rabbit anti-LC3B (Sigma-Aldrich, St. Louis, MO; L7543), polyclonal rabbit anti p62 (PM045), monoclonal rabbit anti-Drp1 (8570), monoclonal mouse anti-parkin (4211), monoclonal mouse anti-Parkin (M230-3) were from MBL International (Woburn, MA, USA); polyclonal rabbit anti-PINK1 (BC100-494) was from Novus Biologicals (Littleton, CO, USA); monoclonal mouse anti-β-tubulin (T7816) was from Sigma-Aldrich (St. Louis, MO, USA); monoclonal mouse anti-actin was from MP Biomedicals (691001; Santa Ana, CA, USA), polyclonal rabbit anti-Mfn1 (sc-50330), was from Santa Cruz Biotechnology (Dallas, TX, USA); and monoclonal mouse anti- Aβ (6E10) (Covance, Emeryville, CA; SIG-39300). Reagents are as follows: chloroquine (CQ; Sigma-Aldrich, C6628), Rapamycin (Rapa; Cayman Chemical, Ann Arbor, MI; 13346) and Aβ (1–42), HiLyte Fluor™ 488-label (Anaspec, Campus Drive Fremont, CA; 60479) carbonyl cyanide m-chlorophenyl hydrazine (Sigma-Aldrich, CCCP; C2759). H2DCFDA (DCF; Sigma D6883).

### Cell lines

The human *APOE3* and *APOE4* expression vectors were used to transfect murine microglial cell line N9. Expression vectors (pCDNA3-flag-APOE2, pCDNA3-flag-*APOE3* or pCDNA3-flag-*APOE4*) were generated using pCMV4-*APOE2*, pCMV4-*APOE3* and pCMV4-*APOE4* addgene expression vectors as template for PCR amplification using the following primers: Forward-kpnI: ggccGGTACCCCAATCACGGCAGGAAGATGAAGG, and Reverse-FLAG-XbaI: ccgTCTAGATTACTTATCGCGTCATCCTTGTAATCCCAGTGATTGTCGCTGGGCACAG. Following stable transfection and selection, the G418-resistant colonies were examined for flag expression, and several clones from each APOE isoform were chosen for further analysis (example for the various clones is presented in Figure S1). The clones were grown in Roswell Park Memorial Institute (RPMI)-1640 medium supplemented with antibiotics and 10% heat-inactivated fetal bovine serum (FBS, Hyclone, CH30160.03). Cells were incubated at 37oC in 5% CO2 in air, and the medium was changed every 3–4 days. Cells were passaged when 70% confluent using trypsin/Di-sodium ethylenediaminetetra-acetic acid (Biological Industries, 03-045-1). Cells were cultured at 30% confluence in growth medium, 1–2 days before each experiment.

### Transfections and expression of N9 microglial *APOE2*, *APOE3*, and *APOE4* isoforms

Transfection was performed using the Transit 2020 reagent (Invitrogen, Carlsbad, CA) as follows: cells were plated (5 × 10^5^ cell/well of a 6-well plate) and transfected 24 h later with 2.5 µg of pCDNA3-flag-*APOE2/APOE3/APOE4* respectively using Transit 2020 reagent containing 1.5 ml medium supplemented with 10% Opti-MEM^®^ for 24 h. Medium was replaced after 24 h.

Transient transfection of *APOE3* and *APOE4* microglia with the tandem LC3-EGFP-mRFP plasmid (Addgene ptfLC3, #21074), was performed using Transit2020 transfection reagent (Invitrogen, Carlsbad, CA), the cells were subjected to autophagy manipulation, and their nuclei were stained with 1 µg/ml Hoechst 33,342 for 10 min. Subsequently, the cells were fixed and microscopically analyzed using Leica TCS SP8 confocal microscope (63x magnification).

### Lysate preparation and immunoblotting

Cells were grown in medium with or without the indicated stimuli. Cells were solubilized in lysis buffer (50 mM HEPES pH = 7.5, 150 mM NaCl, 10% glycerol, 1% triton X, 1 mM EDTA pH = 8, 1 mM EGTA pH = 8, 1.5 mM MgCl_2_, 200 µM Na_3_VO_4_, 150 nM aprotinin, 1 µM leupeptin, 500 µM AEBSF). Lysates were cleared by centrifugation as described [12, 13]. Lysates were resolved by SDS-polyacrylamide gel electrophoresis through 10–15% gels and electrophoretically transferred to nitrocellulose membranes. Membranes were blocked for 1 h in TBST buffer (0.05 M Tris HCl pH 7.5, 0.15 M NaCl and 0.1% Tween 20) containing 6% milk, blotted with primary antibodies for 2 h, followed by secondary antibody linked to horseradish peroxidase for 1 h. Immunoreactive bands were detected with the enhanced chemiluminescence reagent. Densitometric analysis of the results was performed using the ImageJ program.

### In situ Aβ plaques degradation in the presence of *APOE2*,* APOE3* and *APOE4* microglia

This assay is based on the measurement of the ability of the cells to clear insoluble Aβ plaques following their co-incubation with brain sections from 5XFAD mice containing Aβ plaques as previously described [14, 15]. In Brief, to assess the ability of *APOE* expressing microglia to clear Aβ plaques ex vivo, 100,000 N9 microglia cells (in 100 µl medium) expressing different human *APOE* variants were seeded on 20 μm brain section isolated from 18 months old 5XFAD mice. The Brains were snap freeze with liquid nitrogen and kept in -80 C until used. Accordingly, 20 μm coronal frozen brain sections were prepared. The sections were incubated with the *APOE2*,* APOE3* and *APOE4* expressing microglial cells for 48 h at 37 C and then fixed in 4% formaldehyde in PBS for 10 min followed by wash with 0.1% Triton solution for 10 min after which they were incubated with blocking solution (8% Horse Serum, 0.3% Triton, 1 gr/ml BSA, 0.02% Sodium Azide in PBS) for 30 min. Sections were then incubated with primary antibodies anti- Aβ (ab201060, 1:200, Abcam), in a blocking solution for one hour at room temperature. Following three washes with PBS-tween, sections were incubated with Alexa-fluor 488-conjugated goat anti-rabbit (A-11008), 1:500, Invitrogen) in TBS, for 45 min at room temperature, and then developed as previously reported [16]. Slides were observed using a light microscope (Nikon Eclipse 80i) and fluorescent images were taken using a microscope camera (Micropublisher 6, Teledyne Photometrics). The quantification was done using ImageJ software analysis. Analysis of the Aβ plaque burden was performed as percentage of the area covered in plaques using the ImageJ software.

### Quantification of fluorescent Aβ uptake and degradation of *APOE3* and *APOE4* N9 microglia using IncuCyte live cell imaging

*APOE3* and *APOE4* expressing microglial cells were cultured in a 12-wells plate. The cells were treated or untreated with 10 µM chloroquine for 2 h, followed by the addition of 0.1 µM HiLyteTM 488-labeled Aβ_1−42_ for an additional 2 h. Changes in Aβ intensity inside the cells were measured using the IncuCyte machine immediately following the addition of HiLyteTM 488-labeled Aβ, and uptake was measured. Aβ degradation was determined during 2 h following medium replacement and PBS washing. To determine the effect of autophagy inhibition cells were treated with 10 µM chloroquine followed by the addition of 0.1 µM HiLyteTM 488-labeled Aβ 1–42 for 2 h. The changes in Aβ intensity inside the cells were measured using Incucyte machine. Incucyte is an automated microscope that provides a label-free, non-invasive method of monitoring cells at desired time intervals in their native environment, in cell culture incubator. Cells first images were captured immediately post HiLyteTM 488-labeled Aβ treatment, afterwards one image per well was collected every 15 min for the duration of 2 h (4 fields were taken in the well).

### Analysis of mitochondrial network morphology

*APOE2*,* APOE3*, and *APOE4* expressing N9 microglia were incubated with 100 nM MitoTracker™ Deep Red FM (M22426, Invitrogen, Carlsbad, CA, USA) and 1 µg/ml Hoechst 33342 (B2261, Sigma-Aldrich) for 30 min at 37 °C, and fixed in ice-cold methanol for 15 min. (Mitochondrial network morphology of individual cells was visualized by Leica TCS SP8 confocal microscope (63 X magnifications) and analyzed using the ImageJ macro tool MiNa [17].

### Measurement of MMP by flow cytometry

To assess MMP by flow cytometry, as described by others [18–23], the cells were incubated with 100 nM MitoTracker™ Red CMXRox for 30 min at 37 °C. The cells were then trypsinized, washed in PBS, centrifuged, and resuspended in 200 µl PBS. Cell fluorescence was analyzed using Stratedigm S1000EXi Flow Cytometer within 30 min. Analysis of fluorescence levels was performed using the Cyflogic software.

### MTT and cell number measurement

Cell number was determined using the methylene blue assay according to the manufacturer instructions and as previously described. Mitochondrial metabolism was assessed using the [3-(4,5-dimethylthiazol-2-yl)-2,5-diphenyl] tetrazolium bromide (MTT) assay [24–26].

### Statistical analysis

All experiments were performed at least three times. Results are presented as mean ± SE. For Aβ uptake and degradation assay we have analyzed the results obtained by Live-Cell analysis (Incucyte^®^). For the MMP assay we have used the Flow Cytometer as described above to collect the data. Differences between means were assessed by the two-tailed Student’s t test and one-way ANOVA GraphPad Prism for Windows and Microsoft Excel software. Significance was assigned at *p* < 0.05.

## Results

### Insoluble Aβ plaques elimination and soluble Aβ uptake are inhibited in *APOE4* expressing microglia and are affected by chloroquine

**Fig. 1:**
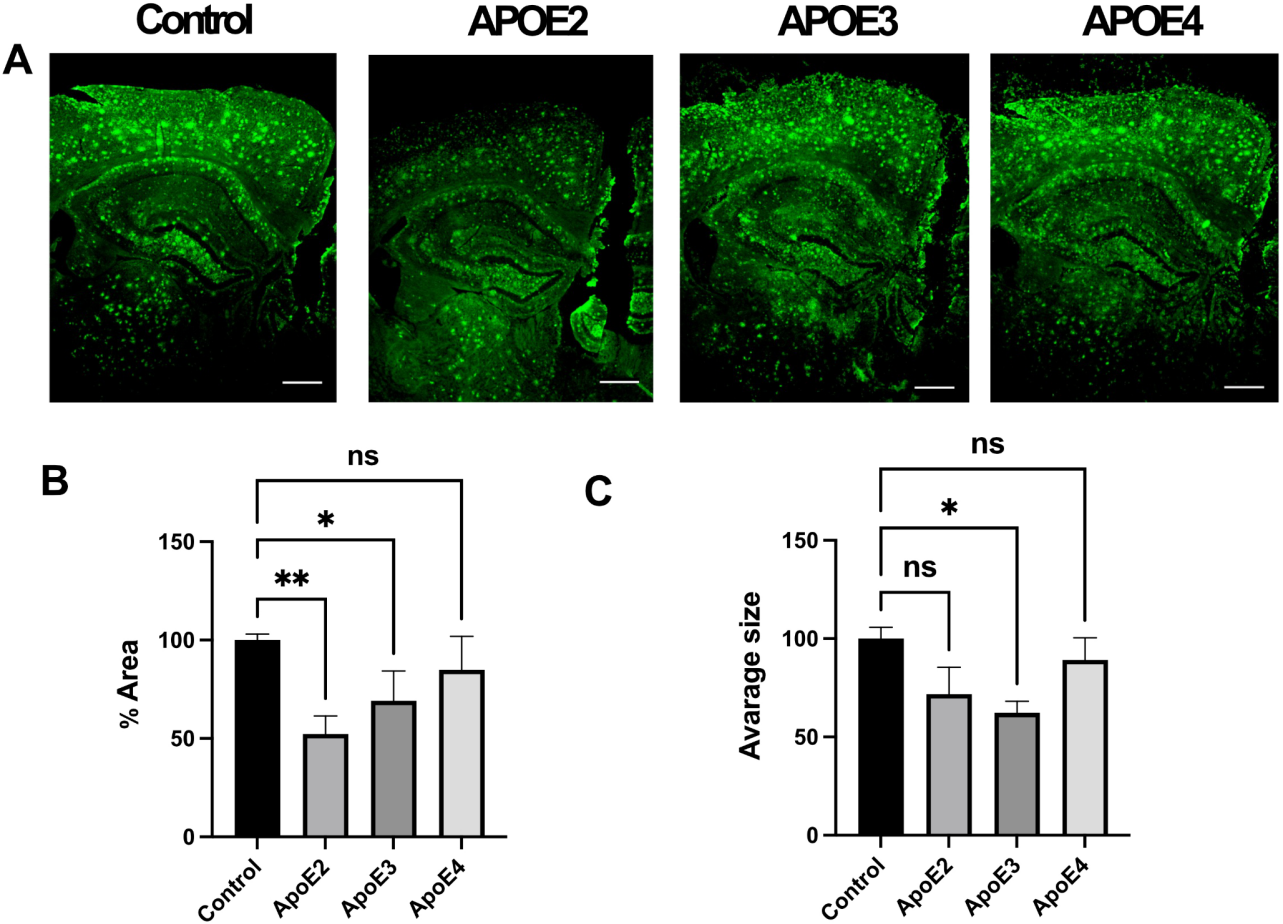
Impaired amyloid plaque clearance by *APOE4* microglia cells. Brain section from 5XFAD mice sections were incubated for 48 h with microglia cells expressing different type of human *APOE*: *APOE*2, *APOE*3 and *APOE*E4. Media without cells was used as a negative control. Following 48 h the brain sections were stained with anti- Aβ antibody against Aβ 1–42. Amyloid plaques clearance in the hippocampus region was assessed by microscope and ImageJ. Scale bar is 500 µM (**A**). Representative images of amyloid plaque-containing hippocampi following incubation with microglia. (**B**) Analysis of the % hippocampus area covered by Aβ plaques from each microglia type or (**C**) average plaques size vs. normalized to control (*n* = 4–5). Statistical analysis was performed using one way ANOVA; * *p* = 0.01, ** *p* < 0.0001

Autophagy plays a role in the degradation of senile plaques [27]. We and others have previously shown that *APOE4* expressing astrocytes have impaired uptake of Aβ. In order to assess the effect of *APOE4* expression in microglia cells on their ability to clear Aβ plaques, compared to *APOE2* or *APOE3* expressing microglia, we performed an in situ plaque removal assay (Fig. 1). In this assay, we measured the different cell lines ability to remove senile plaques from brain sections of 5XFAD transgenic mice, containing high Aβ plaque load. *APOE2* and *APOE3* expressing microglia reduced Aβ plaques level by 48% ± 9.1% (*p* < 0.005 ) and 31% ± 15% (*p* < 0.05) respectively, out of total Aβ plaque area, as compared with the negative control condition containing cell free medium only. In addition, there was a significant reduction in plaques size by 33% ± 7 (*p* < 0.05) following incubation with *APOE3* expressing microglia. However, *APOE4* expressing microglia didn’t show a significant effect on the area covered by Aβ plaques (*p* = 0.5) or the average plaque size (*p* > 0.99), compared to the negative control. Therefore, *APOE2* and *APOE3* microglia seem to be more effective in Aβ plaques clearance as opposed to *APOE4* microglia.

**Fig. 2:**
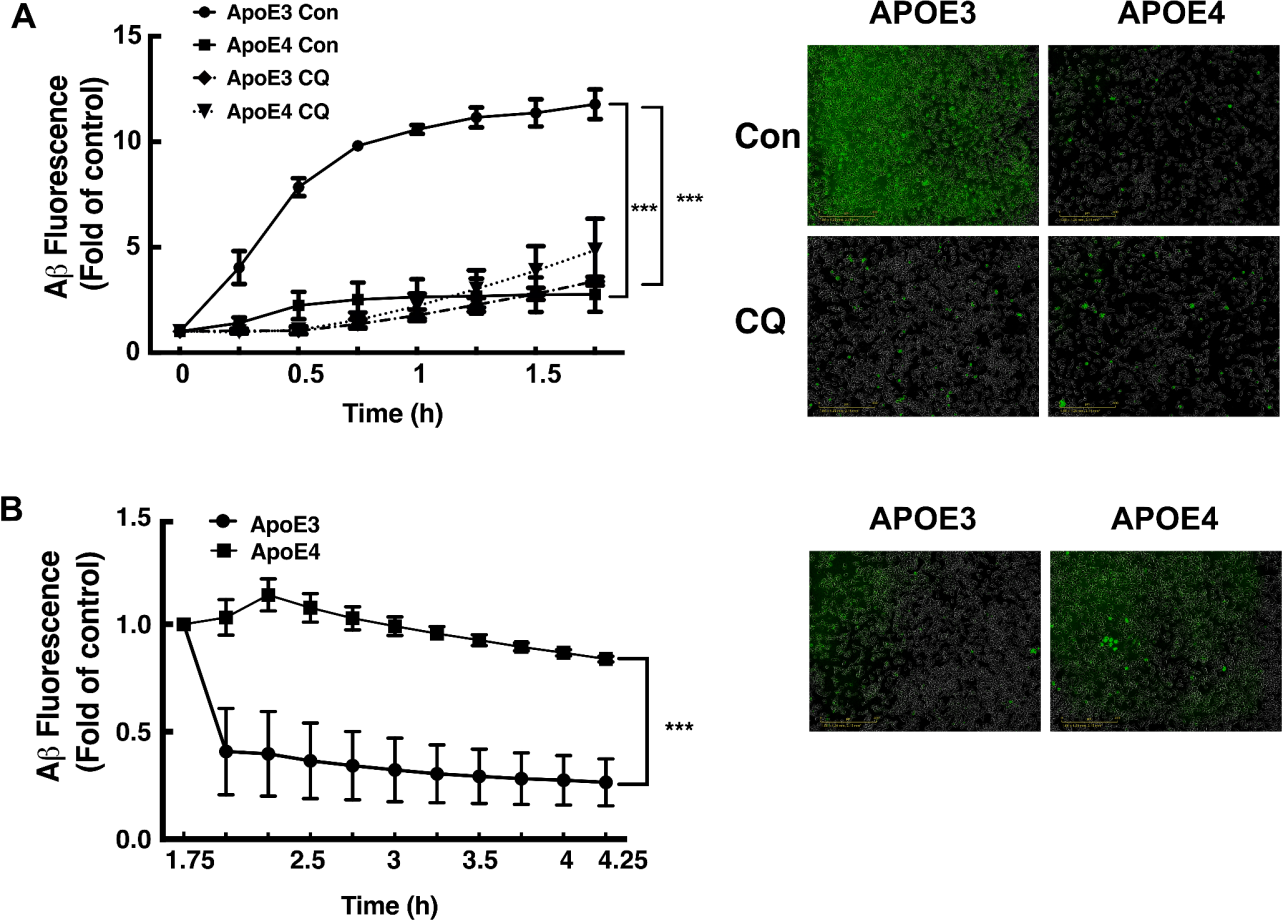
Uptake and degradation of Aβ by *APOE4* compared to *APOE3* microglia. *APOE3* and *APOE4* expressing microglial cells were cultured in a 12-wells plates. Cells were treated for 2 h with 0.1 µM HiLyteTM 488-labeled Aβ 1–42 in the presence or absence of 10 µM chloroquine (CQ). Images of the cells were taken every 15 min, (four fields for each well). To consider the effect of the number of cells in each well, near-infrared (NIR) values were normalized to phase scans.** A** The changes in Aβ intensity inside the cells were measured using Incucyte machine, indicating Aβ uptake. **B** At the indicated time point, cells were washed twice with PBS and incubated with Aβ free medium for further 2 h, in 37◦C. The changes in Aβ intensity inside the cells were measured, indicating Aβ degradation. Right panel depicts representative images in the absence or in the presence of chloroquine (CQ), (scale bars is 400 µM). Left panels depict densitometric analysis of the results which are presented as fold induction of the controls (Mean ± SEM). Statistical analysis was performed using two tailed unpaired student t-test; *** *p* < 0.001, **** *p* < 0.0001 (*n* ≥ 4)

The most common allele of *APOE* is *APOE3* with a frequency of approximately 75% in the general population [28]. Therefore, following the finding that *APOE2* and *APOE3* microglia eliminated Aβ plaques more efficiently than *APOE4* expressing microglia, we next examined the possibility that *APOE4* also affects the uptake of soluble Aβ_1− 42_ compared to APOE3 expressing cells. This was performed by using Live-Cell Analysis (Incucyte^®^) of the amount of fluorescent Aβ, which was accumulated within the *APOE3* and *APOE4* expressing microglia following 1.75 h incubation. As depicted in Fig. 2A, Aβ uptake by *APOE3* cells was significantly higher than the corresponding uptake by *APOE4* cells at various time point measured. Moreover, when cells were co-incubated with chloroquine to inhibit autophagy, the rate of Aβ uptake was significantly reduced in *APOE3* cells, but not in the *APOE4* cells, whose basal rates of Aβ uptake were low to begin with and was not affected by this treatment. Taken together, these findings show that *APOE4* inhibits Aβ plaques elimination and soluble Aβ uptake and suggest that the impairment of autophagy may be related to these effects. Next, we measured the rate of Aβ degradation in *APOE3* compared to *APOE4* cells, and as shown the degradation of Aβ was significantly higher in *APOE3* expressing cells compared to *APOE4* expressing microglial cells (Fig. 2B).

### Autophagy and autophagic flux are inhibited in *APOE4* expressing microglia

**Fig. 3:**
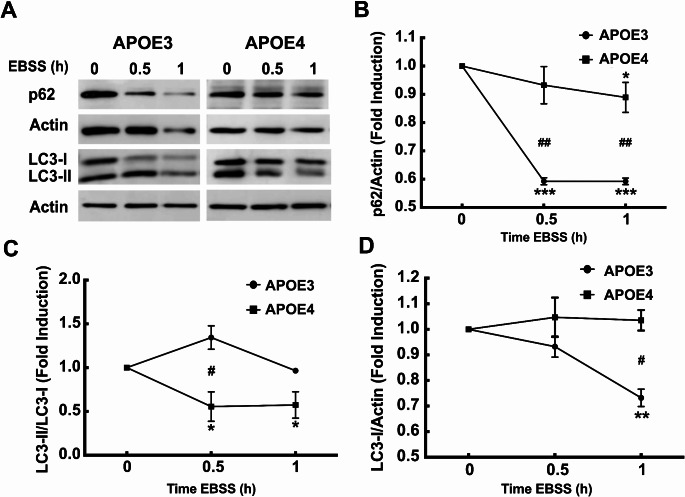
EBSS induced autophagy in *APOE3* and *APOE4* expressing N9 microglia. *APOE4* and *APOE3* microglia were starved (incubation in EBSS) for the indicated times. The levels of p62 and LC3-I and LC3-II were measured by western blot analysis. In B, C and D, the densitometric analyses are presented as fold induction compared to the controls at time = 0 of each of the cell lines (mean ± SE; *p < 0.05, **p < 0.01, *** *p* < 0.001 treated compared to untreated cells; within group; #*p* < 0.05, ## *p* < 0.01 between groups (*APOE3* compared to *APOE4*)

**Fig. 4:**
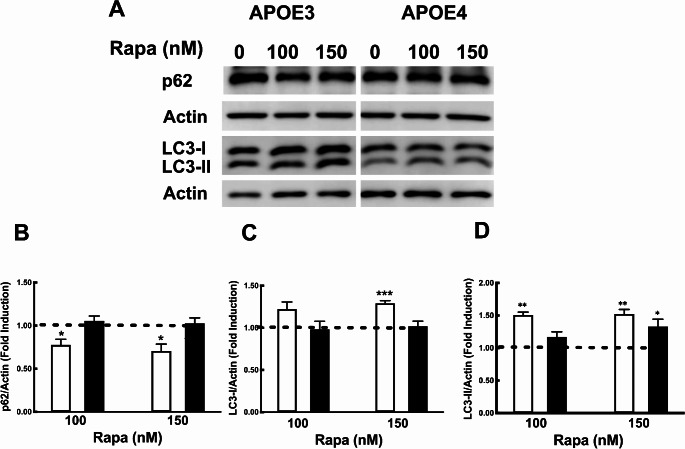
Rapamycin induced autophagy in *APOE3* and *APOE4* expressing N9 microglia. *APOE3* and *APOE4* microglia were treated for 24 h with rapamycin (Rapa) at the indicated concentrations. The autophagic markers p62 and LC3 were measured by western blot analysis. Lower panels depict densitometric analysis of the results which are presented as fold induction compared to the untreated control of each cell line, (mean ± SE; *p < 0.05, **p < 0.01,***p < 0.001).

As shown in Fig. 2, Aβ uptake was inhibited by chloroquine. Previously we and others have also demonstrated that *APOE4* expressing astrocytes confer impaired autophagy [16, 29]. Thus, we next explored the role of *APOE* alleles on autophagy in microglial cells, we examined the extent to which autophagy is differentially regulated in *APOE3* and *APOE4* expressing N9 microglial cells. Hence, we first used the known inducer of autophagy, namely, amino acid deprivation (incubation in EBSS), for the indicated time periods, to determine the expression of the autophagic proteins, LC3 and p62/SQSTM1 (abbreviated as p62) in the *APOE3* and *APOE4* expressing cells. During autophagy LC3-I undergo lipidation (LC3-II), and is associated with the autophagosomal membrane serving as a marker for autophagy [30]. P62, binds LC3 and ubiquitinated proteins marking them for degradation, however, p62 is also degraded during autophagy [31]. Figure 3 shows that the LC3-II/LC3-I ratio, which is a marker of autophagic activation, was significantly higher in *APOE3* microglia compared to *APOE4* microglia, after 0.5 and 1 h of starvation (1.343 ± 0.134 and 0.964 ± 0.005) compared to (0.555 ± 0.167, and 0.573 ± 0.150), respectively. As shown, the levels of LC3I were significantly reduced in *APOE3* expressing cells, peaking at 1 h (0.732 ± 0.033) but not in *APOE4* expressing microglia. Moreover, the levels of p62, which is associated with the degradation steps during autophagy, decreased following amino acid starvation more rapidly in *APOE3* microglia compared to *APOE4* microglia (0.592 ± 0.012 and 0.592 ± 0.011 compared to 0.932 ± 0.065, and 0.889 ± 0.053, respectively). Thus, the results suggest that induction of autophagy may be also impaired in *APOE4* microglia. Next, we examined autophagy levels in *APOE3* and *APOE4* microglia following treatment with the mTOR inhibitor, rapamycin, which is also inducer of autophagy. As shown in Fig. 4, autophagy was significantly lower in *APOE4* microglia following treatment with 100 or 150 nM rapamycin. Accordingly, the LC3II/actin ratio under these conditions was 1.508 ± 0.043 and 1.522 ± 0.069 in *APOE3* expressing microglia, compared to 1.171 ± 0.078 and 1.33 ± 0.112, in *APOE4* expressing microglia, (Fig. 4) whereas the corresponding p62 levels were 0.777 ± 0.063 and 0.706 ± 0.079, compared to 1.055 ± 0.055 and 1.026 ± 0.063, in *APOE4* expressing microglia, which did not show any change in p62 levels during the experimental time frame (Fig. 4).

**Fig. 5:**
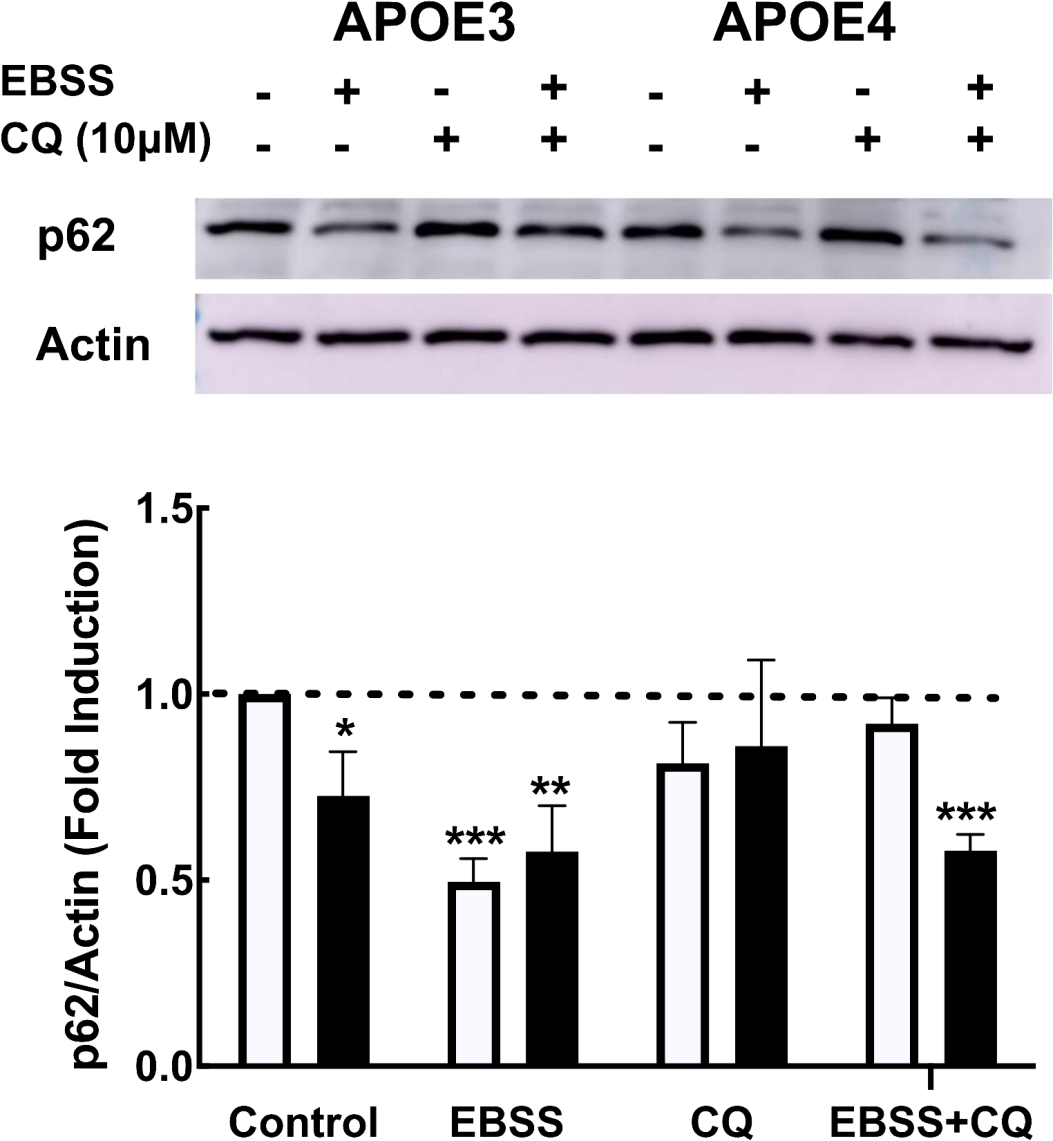
Autophagic flux in *APOE3* and *APOE4* expressing N9 microglia. Cells were starved (incubation in EBSS) for 1 h, with or without 10 µM chloroquine (CQ). The markers of autophagy, p62 (B) and LC3 (C) were measured by western blot analysis. Lower panel depicts densitometric quantification of the results which are presented as fold induction compared to *APOE3* control (mean ± SE; ^*p* < 0.05 and, ^^*p* < 0.01,^^^*p* < 0.001, (EBSS/CQ treated compared to untreated cells); *p < 0.05 **p < 0.001,ApoE3 compared to *APOE4* cells (white bars, *APOE3* and black bars, *APOE4*)

**Fig. 6:**
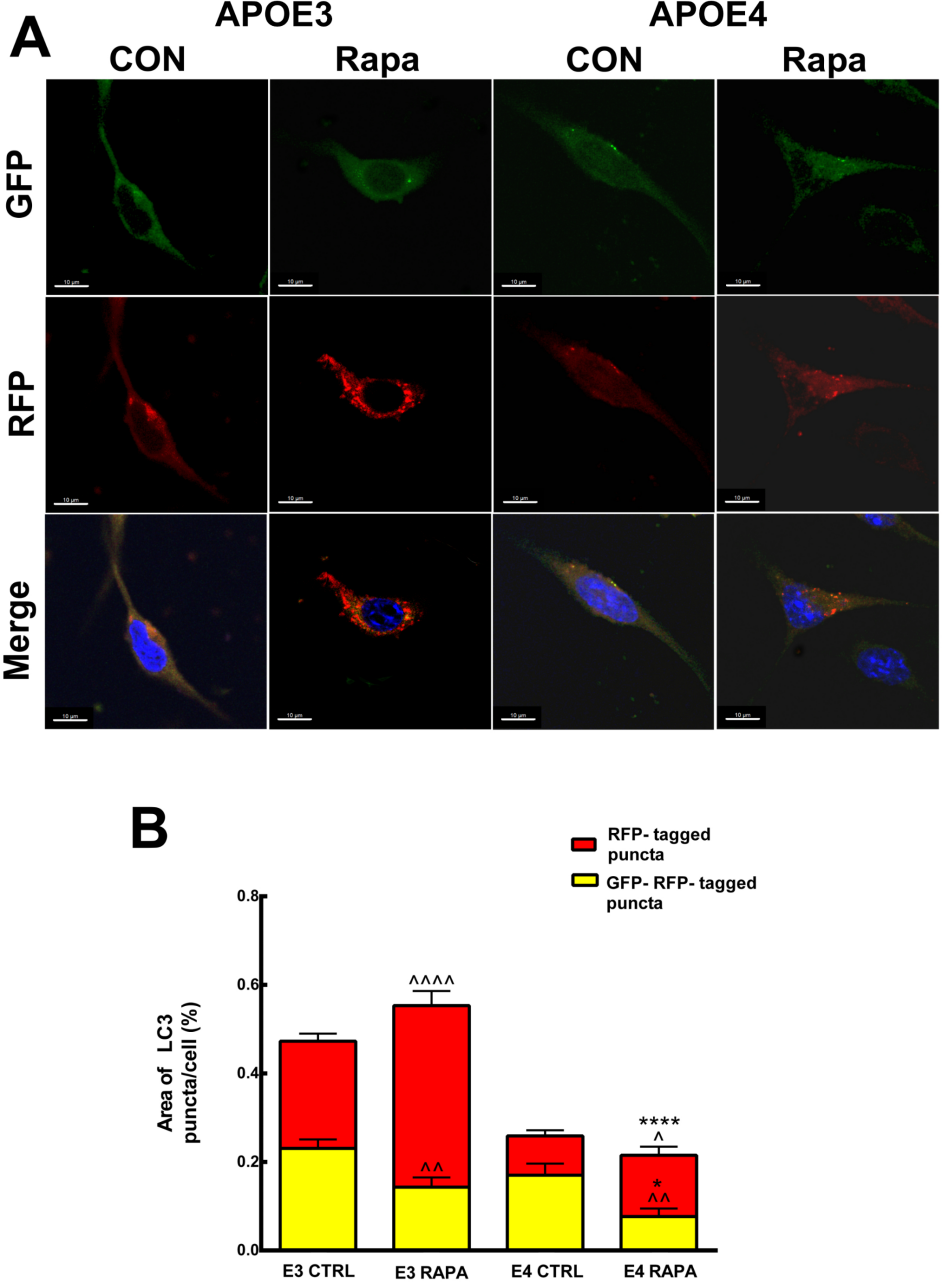
Rapamycin induced autophagy in *APOE3* and *APOE4* N9 microglia. *APOE3* and *APOE4* expressing microglia were transiently transfected with LC3-EGFP-mRFP expression vector. 48 h later, cells were treated or untreated with 150nM rapamycin for further 24 h. Eventually, the cells were (**A**) photographed using LeicaSP8 laser confocal microscopy are shown in the representative images (Scale bars is 10 μm). **B** Quantification of the results was performed using Image pro 10 analysis of the LC3-EGFP-mRFP (yellow) or LC3-mRFP (red) positive puncta area (%); (data are the means ± SE; ^^*p* < 0.01, ^^^*p* < 0.001); treated compared to untreated cells. Means ± SE (****p < 0.001 and *p < 0.05); *APOE3*- compared with *APOE4*. *n* ≥ 50 cells/treatment

Since autophagy is a dynamic process, we next determined the levels of p62 following amino acid starvation as autophagy inducer, in the presence or in the absence of chloroquine, which inhibits fusion of the autophagosome with the lysosome. As can be seen in Fig. 5, EBSS led to decrease in p62 levels following 1 h starvation, in *APOE3* and *APOE4* microglia, which was inhibited by chloroquine treatment. However, the levels of p62 following chloroquine treatment were significantly higher in *APOE3* microglia compared to *APOE4* microglia (0.954 ± 0.059 and 0.629 ± 0.068, respectively). Furthermore, we used the tandem LC3-GFP-RFP reporter [32], to monitor autophagy. We found that following rapamycin treatment, 41.03% ± 3.268 of autophagosomes were fused with the lysosomes (RFP-tagged red puncta), compared to 13.84% ± 1.96 in the *APOE4* expressing microglia (Fig. 6). Thus, autophagosomes delivery to lysosomes is hampered in *APOE4* microglia. Taken together, these results show that *APOE4* reduces basal and the induced autophagy in N9 microglia cells.

### *APOE4* expression affects cell viability and mitochondrial activity

**Fig. 7:**
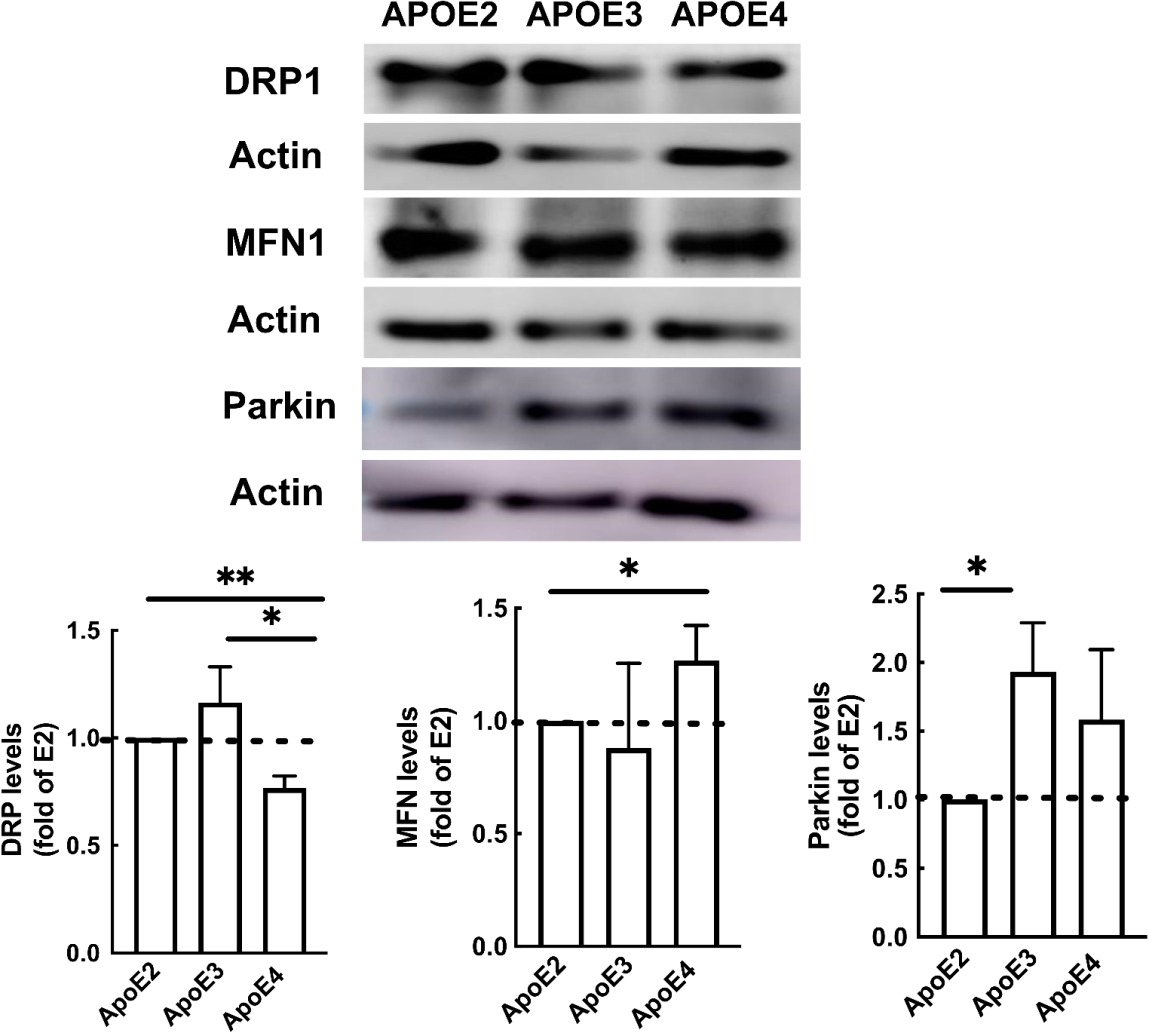
Levels of mitochondrial dynamics proteins in *APOE3* and *APOE4* expressing N9 miroglia. *APOE2*, *APOE3* and *APOE4* microglia were subjected to Immunoblot, using the indicated antibodies. Upper panels, representative results; lower panels, densitometric analysis of *APOE4* or *APOE3* cells as fold of *APOE2*. The results are the means ± SE; *p < 0.05 and **p < 0.01 of at least four experiments

Previously we investigated the effect of *APOE4* expression on several aspects of mitochondrial function and dynamics in astrocytes [33]. We showed that in astrocytes, *APOE4* expression is linked to altered mitochondrial dynamics and impaired mitochondrial function. In order to examine the effect of *APOE* expression in microglia, we next examined the expression of fission, fusion and mitophagy proteins in microglia expressing human *APOE2*,* APOE3* or *APOE4* (Fig. 7). As shown, *APOE4* microglia exhibit lower Drp1 levels (fission) compared to *APOE2* and *APOE3* expressing microglia, higher Mfn1 levels (fusion) in *APOE4* compared to *APOE2* and increased parkin levels in *APOE3* compared to *APOE2* expressing microglia. These results imply that *APOE4* microglia may exhibit reduced fission and altered mitochondrial dynamics.

**Fig. 8:**
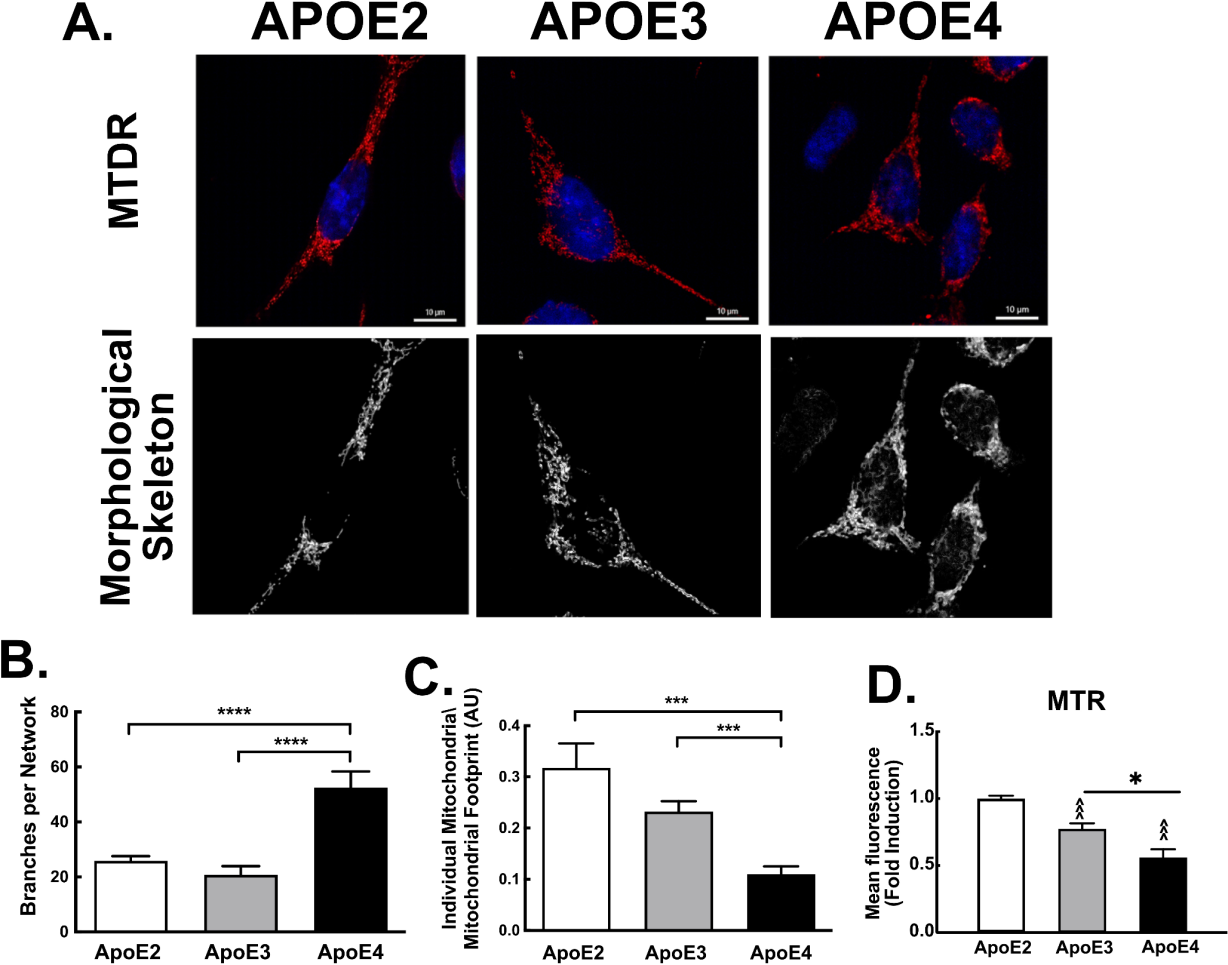
Mitochondrial morphology in *APOE* isoforms expressing microglia. *APOE2*, *APOE3* and *APOE4* microglia were incubated with MitoTracker Deep Red (MTDR). Upper panels, representative images and morphological skeleton analysis as generated by the MiNa tool (Scale bars, 10 μm) (**A**) Lower panels, quantification of branches per network (mean network size) and individual mitochondria relative to the mitochondrial network footprint (**B-C**). (*n* ≥ 50 cells) results are presented as the mean ± SE (***p < 0.001 and **** *p* < 0.0001). **D**. Cells were stained with MitoTracker Red (MTR) and fluorescence was measured (*APOE3* compared to *APOE4*; *p < 0.05 and *APOE2* compared to *APOE3* or *APOE4*; ^^^*p* < 0.001)

To further investigate whether *APOE4* is related to decreased mitochondrial fission and degradation, we studied the mitochondrial network morphology using MitoTracker Deep Red staining. As demonstrated (Fig. 8) *APOE4* expressing microglia exhibited significantly higher levels of branches per networks (52.455 ± 5.905) compared to the *APOE2* (25.837 ± 1.724), and *APOE3* (20.774 ± 3.118). Furthermore, *APOE4* expressing microglia exhibited significantly lower levels of individuals mitochondria (0.109 ± 0.015) compared to *APOE2* expressing microglia (0.317 ± 0.047), and to *APOE3* expressing microglia (0.232 ± 0.02), isoforms respectively, the mitochondrial network of the *APOE4* microglia is indeed more hyperfused; (exhibits more branching and less individual mitochondria). The reduced fission/mitophagy observed in the *APOE4* microglia may indicate abnormal metabolic state or may represent mitophagy impairment.)

**Fig. 9:**
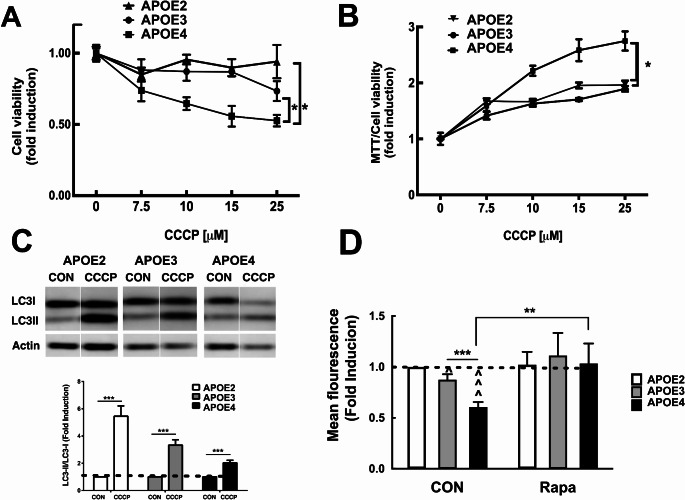
Mitochondrial function in *APOE2*, *APOE3* and *APOE4* N9 microglia. *APOE2*, *APOE3* and *APOE4* microglia were incubated for 4 h with CCCP at the indicated concentrations. Cells were subjected to MTT and Methylene blue (MB) assays. **A** represent cell viability (MB) and **B** represent MTT assay normalized to MB (viability). The results are presented fold of the untreated controls (mean ± SEM). Statistical analysis was performed using 2way-ANOVA. * *p* < 0.05, between groups, comparison between *APOE2*, *APOE3* or *APOE4* expressing N9 cells, (*n* ≥ 3). (**C**) Cells were treated for 4 h with 15 µM CCCP. Levels of LC3 were determined by immunoblot upper panel, lower panel depicts densitometric analysis of the results which are presented as fold induction compared to the untreated control of each cell line (mean ± SE, ***p < 0.001), treated compared to untreated cells. **D***APOE2*, *APOE3*, and *APOE4* expressing microglia were incubated with 150 nM rapamycin for 24 h. Then cells were stained with MitoTracker Red (MTR). Fluorescence intensities were measured using flow cytometry. Results are presented as the fold induction of the mean fluorescence of *APOE3* control. (mean ± SE, ^*p* < 0.05, ^^^^*p* < 0.0001. *APOE3* compared to *APOE4* cells; ***p < 0.001. APOE4 treated compared to APOE4 untreated **p < 0.01). n *≥* 4

Next, we examined the effect of mitochondrial damage induced by mitochondrial uncoupler CCCP [34] on cell viability and mitochondrial activity. We found that following CCCP treatment, cell viability of *APOE4* expressing microglia was significantly lower compared to *APOE2* and *APOE3* expressing cells (Fig. 9A). Nevertheless, mitochondrial activity as estimated by MTT assay, was significantly higher in *APOE4* expressing microglia may be as a compensation mechanism (Fig. 9B). Next, we examined the effect of CCCP treatment on autophagy. As shown in Fig. 9C, CCCP treatment significantly enhanced autophagy in *APOE2*, *APOE3* and *APOE4* expressing microglia though the effect was significantly lower in *APOE4* expressing cells.

### Rapamycin induces mitophagy and repairs mitochondrial function in *APOE4-*expressing microglia

We next examined whether non-toxic autophagy/mitophagy induction could reduce mitochondrial dysfunction in *APOE4* microglia. As shown in Figs. 4 and 6, rapamycin treatment increased autophagy in *APOE4* expressing cells. Mitochondrial membrane potential (MMP) is a crucial aspect of mitochondrial function, reflecting the health and activity of mitochondria. We next examined the MMP in the different APOE-expressing N9 microglia following rapamycin treatment, by flow cytometry (Fig. 9D). The results obtained using MitoTracker Red fluorescent dye (MTR), which stains mitochondria in an MMP-dependent manner, indicate that compared to *APOE2* as a control, *APOE4* expressing microglia exhibited reduced MMP levels (0.606 ± 0.048) and so did *APOE3* expressing microglia (0.874 ± 0.052). Following rapamycin treatment *APOE4* expressing microglia exhibited significantly increased MTR levels (1.033 ± 0.196) compared to the untreated *APOE4* microglia. Taken together, these results demonstrate that rapamycin may correct mitochondrial function in the *APOE4* microglia.

## Discussion

Degradation of cellular constituents including dysfunctional organelles and protein aggregates occurs mainly by the process of autophagy [35]. The autophagic process is characterized by the formation of double-membrane vesicles -autophagosomes, which deliver the degraded material to the lysosomes [7]. Autophagy was previously linked to neurodegenerative diseases including AD [27, 36–45]. Also, a connection between apoE genotype and autophagy was demonstrated in astrocytes [8, 16, 46]. In the present study we used N9 murine microglia cells that express apoE isoforms to examine their effects on autophagy and Αβ uptake and degradation. It was previously shown that both astrocytes and microglia can clear Aβ [14, 47, 48] and it was suggested that *APOE4* confers AD risk through impaired Aβ clearance compared to *APOE3* and *APOE2* [16, 49]. It was previously suggested that APOE4 impairs microglia maturation by affecting TGF-beta signaling [50] Yet APOE4 role in microglia autophagy was not reported. Here, we show that *APOE4* expressing microglial cells have impaired ability to clear Aβ plaques from 5XFAD brain sections. Moreover, *APOE4* cells have impaired uptake and degradation of Aβ. Interestingly, the uptake was inhibited by chloroquine treatment which inhibits fusion of autophagosomes with the lysosomes. To monitor autophagy we determined the levels of LC3 and p62 proteins. We found that microglia expressing *APOE4* exhibit lower autophagic flux compared to those expressing *APOE3*, following autophagy-inducing treatments. Of note, the effect of APOE4 on autophagy might also be through TGF-beta signaling, as previously suggested [50]. Furthermore, these findings were in agreement with studies showing that *APOE4* expressing astrocytes have reduced autophagy compared to *APOE3* [16]. The interaction between *APOE4* and autophagy has been reported in astrocytes [8, 16], and there are evidence that the lysosomes are also affected by *APOE4*. Accordingly, *APOE4* increases the accumulation of Aβ42 in lysosomes leading to cellular toxicity [51, 52]. In addition, endocytosis was shown to be isoform-dependent, with *APOE3* more efficiently facilitating Aβ degradation than *APOE4* [53]. Previous findings, together with our results may suggest a link between the impaired autophagy in *APOE4* microglia, and the impaired uptake of Aβ, since endocytosis and autophagy share similar machinery [54, 55].

Recently it was found the *APOE4* astrocytes have altered mitochondrial dynamics compared to *APOE3* expressing astrocytes [33]. In the present study we examined the mitochondrial dynamics in N9 microglia that express the various APOE isoforms. We found that *APOE4* expressing cells express lower levels of Drp1, compared to *APOE2* and *APOE3* expressing cells. We also observed hyperfused mitochondria in *APOE4* expressing microglia. Following CCCP treatment *APOE4* expressing cells were more susceptible to the treatment as reflected by lower cell viability but with higher mitochondrial activity which might represent compensatory mechanism. To further determine the effect of mitochondrial stress by CCCP we determined the levels of LC3II following CCCP treatment. As shown CCCP enhanced autophagy in all three APOE expressing microglia with lower efficacy in *APOE4* expressing cells. Next, we examined the effect of autophagy/mitophagy induction by rapamycin treatment on *APOE4* microglia mitochondrial dysfunction, our results suggest that rapamycin partially improved APOE4 microglia mitochondrial function.

Taken together the results presented suggest that the pathological effects of *APOE4* in microglia may be mediated by impaired autophagy and by the associated impaired ability of the cells to remove Aβ plaques. The availability of pharmacological agents such as rapamycin, which activate autophagy, thus provides a novel approach to the treatment of apoE4-related brain pathology in AD.

## Electronic supplementary material

Below is the link to the electronic supplementary material.

Supplementary Material 1

## Data Availability

No datasets were generated or analysed during the current study.
