# Supplementary material for: APOE4 impairs autophagy and Aβ clearance by microglial cells
Source: Inflamm Res. 2025 Apr 1;74(1):61. doi: 10.1007/s00011-025-02016-5 (PMC11958439; doi:10.1007/s00011-025-02016-5)
Supplement: Supplementary file 1 — Supplementary Material 1 [file 11_2025_2016_MOESM1_ESM.pdf]

Supplementary

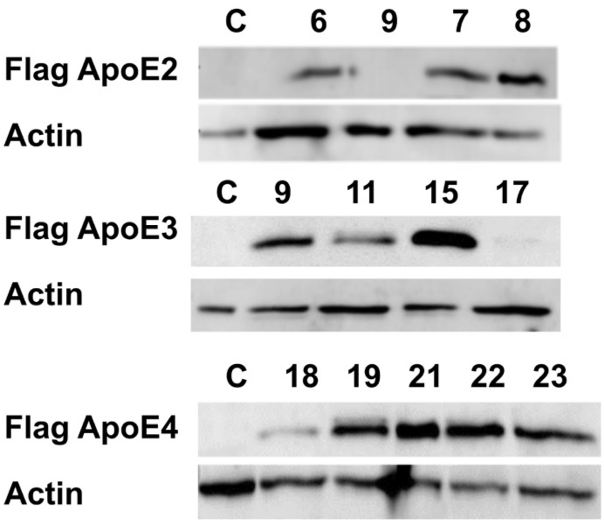

Figure S1 legend

Example for N9 clones stably expressing Flag-*APOE2*, Flag-*APOE3* or Flag-*APOE4*.

N9 cells were transfected with either pCDNA3–Flag-*APOE2*, pCDNA3–Flag-*APOE3 or* pCDNA3–Flag-*APOE4* expression vectors. Following G418 selection selected positive clones were isolated and analyzed by western blot to identify the positive clones. Selected positive cloned with similar expression levels were used for further analysis.
